# Regulation of the Rhythmic Emission of Plant Volatiles by the Circadian Clock

**DOI:** 10.3390/ijms18112408

**Published:** 2017-11-13

**Authors:** Lanting Zeng, Xiaoqin Wang, Ming Kang, Fang Dong, Ziyin Yang

**Affiliations:** 1 Key Laboratory of South China Agricultural Plant Molecular Analysis and Genetic Improvement & Guangdong Provincial Key Laboratory of Applied Botany, South China Botanical Garden, Chinese Academy of Sciences, Xingke Road 723, Tianhe District, Guangzhou 510650, China; zenglanting@scbg.ac.cn (L.Z.); xqwang@scbg.ac.cn (X.W.); mingkang@scib.ac.cn (M.K.); 2 University of Chinese Academy of Sciences, No. 19A Yuquan Road, Beijing 100049, China; 3 Department of Food, Guangdong Food and Drug Vocational College, Longdongbei Road 321, Tianhe District, Guangzhou 510520, China; dongfangxyz@163.com

**Keywords:** biosynthesis, circadian clock, emission, plant volatile, rhythm, substrate, transcription

## Abstract

Like other organisms, plants have endogenous biological clocks that enable them to organize their metabolic, physiological, and developmental processes. The representative biological clock is the circadian system that regulates daily (24-h) rhythms. Circadian-regulated changes in growth have been observed in numerous plants. Evidence from many recent studies indicates that the circadian clock regulates a multitude of factors that affect plant metabolites, especially emitted volatiles that have important ecological functions. Here, we review recent progress in research on plant volatiles showing rhythmic emission under the regulation of the circadian clock, and on how the circadian clock controls the rhythmic emission of plant volatiles. We also discuss the potential impact of other factors on the circadian rhythmic emission of plant volatiles.

## 1. Introduction

Plants synthesize and emit a variety of volatile organic compounds, which have ecological or physiological functions [1,2,3,4,5,6]. In response to external factors, plants emit volatiles “on demand”. For example, upon exposure to abiotic or biotic stress, the vegetative parts of plants emit volatiles that can reduce the negative effects of stress. Emitted volatiles have been shown to increase resistance to high temperature and oxidative stresses, directly defend against herbivores, attract enemies of herbivores, and participate in within-plant or plant-to-plant signaling [7]. In most cases, volatiles show peak emission within a certain period after plants perceive the stress [3,4,5,7], but rarely show a rhythmic emission pattern. However, most floral parts emit volatiles even in undamaged conditions [2]. The emitted floral volatiles play diverse roles in pollination biology [8], and show not only peak emission patterns, but also rhythmic emission patterns in some cases [9]. Rhythmic emission patterns are directly controlled by external factors such as light, or by the endogenous circadian clock.

The circadian clock is a biological oscillator that maintains a 24-h rhythm under normal environmental conditions [10]. Circadian systems are widespread endogenous mechanisms that allow organisms to time their physiological processes in response to predictable day/night cycles [11]. The growth rate of plants is modulated by numerous environmental factors such as light, temperature, and nutrient availability, as well as by endogenous factors such as the developmental stage and hormone signals. Some of these factors are under regulation by the circadian clock [10]. Many vascular plants display a diurnal growth rhythm [12], and in some cases, the rhythm is maintained either under continuous light or dark conditions. Recent studies have partly clarified how the circadian clock regulates the various processes and how these interactions specify the timing of growth under both light/dark and constant environmental conditions [10,11,13,14]. A number of recent studies have shown that plant volatiles can be rhythmically emitted under the control of the circadian clock, and have illustrated the related underlying regulation mechanisms. In this review, we summarize the current knowledge on these topics, and discuss the approaches and criteria that are widely used to study and define the circadian rhythmicity of plant volatiles emission. We also discuss the effects of other factors on circadian rhythmic emission, and propose directions for future research.

## 2. Techniques for the Collection and Detection of Plant Volatiles

**Figure 1:**
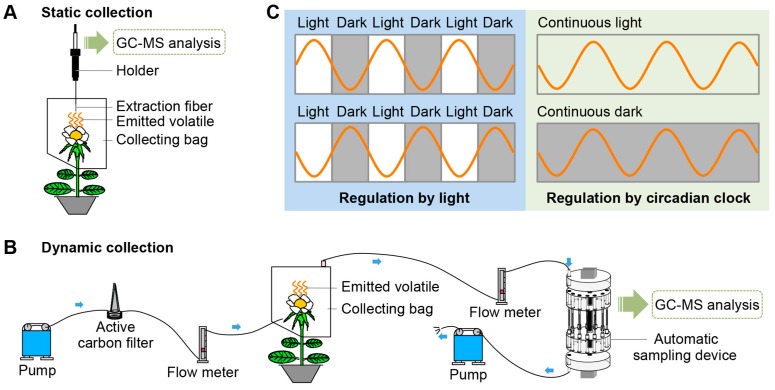
(**A**,**B**) Techniques for the collection and detection of volatiles emitted from plants. After collection, emitted volatiles are separated and analyzed by gas chromatography-mass spectrometry (GC-MS). (**A**) Static collection by solid phase microextraction (SPME); (**B**) dynamic collection using dynamic automatic sampling system. Airflow is controlled by two pumps and is filtered through an active carbon filter. Blue arrows indicate airflow direction; (**C**) rhythmic emissions of volatiles under direct light regulation and under control of the endogenous circadian clock.

Once released into the air, volatiles move freely and are difficult to collect. In addition, the emission of many volatiles is variable. Therefore, to precisely analyze emitted volatiles, techniques to collect these compounds should be effective and efficient. Here, we summarize two widely used collection methods (Figure 1A,B). The combination of solid phase microextraction (SPME) and gas chromatography-mass spectrometry (GC-MS) analysis is very fast, effective, and simple (Figure 1A). The sampling time, temperature, and sample volume influence the effectiveness of SPME extraction [15]; therefore, this method is less suitable for quantitative analyses. To ensure the reproducibility of results, it is necessary and crucial to maintain consistent environmental conditions. Compared with SPME, which is a static sampling technique, the dynamic headspace sampling technique is more suitable for quantitative analyses and for detecting variations in emitted volatiles [15,16]. The dynamic sampling system is composed of two pumps, two flow meters, an active carbon filter, an automatic sampling device, and adsorbents (Figure 1B). Tenax and Porapak Q are the most widely used adsorbents. In this sampling system, airflow controlled by the pump makes the extraction more complete and the results more precise, avoiding regional differences. This method is also suitable for real-time qualitative or quantitative studies.

Studies using these new and sophisticated sampling techniques have markedly increased our understanding of the rhythmic emission of plant volatiles in recent years. Rhythmic emission occurs under direct regulation by light or by the endogenous circadian clock (Figure 1C) [9]. The emission of volatiles is variable and diverse. Some volatiles show a diurnal emission pattern with larger amounts emitted during the day, while others are emitted primarily at night (Figure 1C). To maximize reproductive success, diurnally pollinated flowers tend to maximize volatiles emission during the day, whereas flowers pollinated at night show a nocturnal rhythmicity [17,18,19]. These two emission patterns can be induced either by light or darkness, i.e., by the presence or absence of light. Besides light-induced emission cycling, the emission of some volatiles can be modulated by the endogenous biological clock. This rhythmic emission of volatiles follows a free-running cycle that is independent of environmental conditions and matches a 24-h light/dark period. Additionally, the cycle also continues under continuous dark or continuous light, confirming the circadian nature of the emission (Figure 1C). There are three established criteria for circadian rhythmicity: it should show a periodical circadian oscillation, remain steady under constant conditions during a 24-h period, and a phase-shift should be induced by a change in the daily light/dark cycle [20,21,22]. The ability to collect volatiles continuously and automatically using the automatic headspace sampling system (Figure 1B) has allowed for real-time, convenient, and effective detection of rhythmically emitted volatiles.

## 3. Plant Volatiles Showing Circadian Rhythmic Emission

To date, more than 1700 volatile compounds have been isolated from more than 90 plant families, containing almost 1% of all plant specialized metabolites identified so far [2]. During recent decades, many studies have explored the multi-functional roles of plant volatiles. For environmental adaptation, plants constantly release a wide spectrum of volatile compounds to communicate and interact with various members of their surrounding community, such as conspecific neighboring plants, pollinators, herbivores, carnivorous arthropods, or even parasitoids [2,23,24,25,26]. In some cases, plant volatiles are produced as part of the defense strategy against biotic and abiotic stresses [4,5,7,27]. They can function as both direct and indirect defenses, deterring feeding, and reducing the fecundity and survival rate of the invaders as well as attracting enemies of herbivores [7,28]. They also play critical roles in plant reproduction. In nature, all organisms are under selective pressure to maximize their reproductive success, and plants are no exception. Numerous volatile compounds ultimately ensure the reproductive and evolutionary success of plants by attracting pollinators and seed dispersers [2,23]. Plant volatiles are low-molecular weight compounds with a high vapor pressure at ambient temperatures [1]. During the last several years, it was popularly thought that maybe all plant volatile communication occurred through simple diffusion. The physical properties of volatiles allow them to cross cellular membranes freely and evaporate into the surrounding environment when there are no barriers to diffusion [1]. Recently, however, there was a study demonstrating that passage of volatiles across the plasma membrane relies on active transport in petunia [29].

Volatiles emission is highly regulated, and is restricted to specific times of the day in many plant species. Several recent studies have significantly advanced our knowledge of rhythmic emissions. Table 1 summarizes the volatiles rhythmically emitted from various plant species. Emission levels of some volatiles are also organized in Table 1. According to their different biosynthetic pathways, plant volatiles can be classified into three groups: volatile terpenes, volatile phenylpropanoids/benzenoids (VPBs), and volatile fatty acid derivatives [2,23]. While many studies have reported on the rhythmic emission of volatile terpenes and VPBs (see Table 1), few have investigated whether volatile fatty acid derivatives are emitted in a rhythmic manner under the regulation of the circadian clock. Generally, volatile fatty acid derivatives are involved in the relationships between plants and herbivores, evolving primarily for stress defense [30], whereas volatile terpenes and VPBs are involved in relationships between plants and both pollinators and herbivores, acting as signaling factors for pollinator attraction [31,32]. The activity of the respective pollinators often results in rhythmic volatiles emission [33,34,35]. Usually, emission levels are highest when the primary pollinator is active during the day/night cycle, that is, rhythmic emission allows plants to conserve precious carbon and energy during periods when the pollinator is inactive [23]. The emission of some volatiles from different plant species follows a free-running cycle regulated by the circadian clock (Table 1). In a detailed quantitative time-course analysis of the emission of methyl benzoate from flowers of snapdragon (*Antirrhinum majus* cv. Maryland True Pink), petunia (*Petunia hybrida* cv. Mitchell), and tobacco (*Nicotiana suaveolens*) during two normal light/dark cycles, maximum emission was detected in the three different species during a 24-h period, showing a rhythmic fluctuation [36,37]. Similarly, isoprene emissions from both oil palm (*Elaeis guineensis*) and Grey poplar (*Populus × canescens*) were shown to be under strong circadian control [38,39]. Different volatile compounds in the same plant species can be under similar regulation by the circadian clock. For example, the emission of β-ionone and methyl benzoate from petunia cv. Mitchell flowers was shown to be regulated by a free-running internal circadian clock [36,40]. The endogenous regulator was also shown to control the emission of one VBP (methyl benzoate) and two monoterpenes (myrcene and (*E*)-β-ocimene) from snapdragon flowers [36,41].

## 4. Mechanisms of Circadian Rhythmic Emission of Plant Volatiles

**Figure 2:**
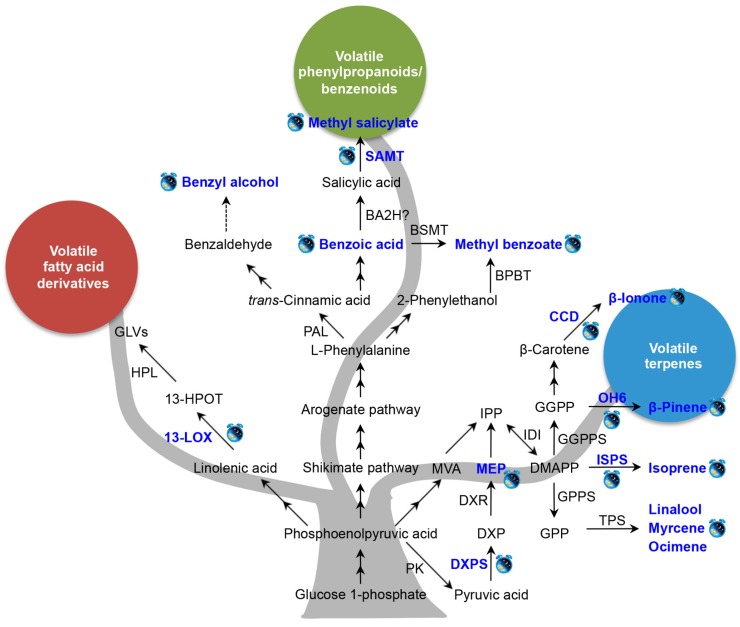
Known factors controlling the circadian rhythmic emission of plant volatiles. Blue text and clock labels indicate that the levels of volatiles emission, intermediate metabolites content, gene transcript, or enzyme activity are under circadian control. Solid arrow is representative of a known route, dotted arrow is representative of an unproven route, and two-way arrow is representative of multiple enzymatic steps. BA2H, benzoic acid-2 hydroxylase; BPBT, benzoyl-CoA:benzylalcohol/2-phenylethanol benzoyltransferase; BSMT, benzoic acid/salicylic acid carboxyl methyltransferase; CCD, carotenoid cleavage dioxygenase; DMAPP, dimethylallyl diphosphate; DXP, 1-deoxy-d-xylulose-5-phosphate; DXPS, 1-deoxy-d-xylulose-5-phosphate synthase; DXR, 1-deoxy-d-xylulose-5-phosphate reductoisomerase; GGPP, geranylgeranyl diphosphate; GGPPS, geranylgeranyl diphosphate synthase; GLVs, green leaf volatiles; GPP, geranyl diphosphate; GPPS, geranyl diphosphate synthase; 13-LOX, 13-lipoxygenase; MEP, methylerythritol phosphate; MVP, mevalonic acid; HPL, hydroperoxide layse; 13-HPOT, 13*S*-hydroperoxy-(*9Z*,*11E*,*15Z*)-octadecatrienoic; SAMT, salicylic acid carboxyl methyltransferase; IDI, isopentenyl diphosphate isomerase; IPP, isopentenyl diphosphate; ISPS, isoprene synthase; TPS, terpene synthase; PAL, phenylalanine ammonia lyase; PK, pyruvate kinase. The rhythmic emissions of methyl benzoate [36], benzyl alcohol [37], methyl salicylate [37,42], isoprene [38,39], β-ionone [40], mycrene [41], and ocimene [41], β-pinene [43], linalool [44], were under circadian control. The content of benzoic acid [36] and MEP [45] was under rhythmic change. ISPS [39], CCD [40], OH6 [43], SAMT [42], DXPS [45], 13-LOX [46] and were under circadian control.

The rhythmic release of volatile compounds under circadian control has been demonstrated in numerous plant species, and several studies have tried to elucidate the mechanisms responsible for rhythmic emission. The known factors controlling the circadian rhythmic emission of plant volatiles are summarized in Figure 2. The details are demonstrated into three biosynthetic pathways. In Figure 2, the blue text and clock labels indicate circadian rhythm. For emitted volatiles or intermediate metabolites, the symbols mean their emission or content levels are under circadian control, whereas for genes, the symbols indicate their gene transcript levels or enzyme activity display a circadian rhythm. In the pathway of volatile fatty acid derivatives, only one upstream gene, *lipoxygenase* (*LOX*), has been linked to circadian regulation. Compared with volatile fatty acid derivatives, there are more studies investigating the underlying mechanisms of circadian rhythmic emission of volatile terpenes and VPBs (Figure 2). In conclusion, there are three main regulation mechanisms of the circadian rhythmic emission of plant volatiles: gene expression of key or final enzymes in biosynthetic pathways or their enzyme activity, substrate availability, and transcription factors.

Several studies have shown that the rhythmic expression of genes encoding enzymes involved in the final steps of volatiles biosynthesis play a role in circadian control. When *Artemisia annua* was cultivated under constant light or constant dark conditions, *QH6* (a β-pinene synthase gene) transcript levels continued to fluctuate with a circadian rhythm, resulting in a diurnal pattern of β-pinene content and emission [43]. In petunia, the maximum emission of β-ionone from flowers during daylight was shown to be in step with the expression of *PhCCD1*, which encodes carotenoid cleavage dioxygenase (CCD), the enzyme responsible for β-ionone formation. This result indicated that circadian modulation of *PhCCD1* controlled β-ionone emission [40]. Salicylic acid carboxyl methyltransferase (SAMT) catalyzes the conversion of salicylic acid (SA) into methyl salicylate (MeSA). In *Stephanotis floribunda*, the expression level of *SAMT* showed a rhythmic oscillation that coordinated with the emission of MeSA from flowers, while the SAMT protein levels did not show rhythmic changes [42]. A similar phenomenon was observed for isoprene emission from Grey poplar, in which the gene encoding the isoprene biosynthesis enzyme, *isoprene synthase* (*ISPS*), showed circadian variations in its transcript levels [39]. However, the ISPS protein content did not correlate well with isoprene emission [39]. Enzyme activity may also influence the circadian rhythm. For example, SAMT activity oscillations were observed in *Stephanotis* flowers, and peak activity coincided with nocturnal MeSA emission [42]. These studies clearly demonstrate that for some volatiles, the rhythmic regulation of their emission occurs both at the transcriptional and post-translational levels.

The biochemical and molecular pathways of volatiles formation are complex, and involve the interplay of several biochemical pathways and hundreds of genes [6]. The expression of key genes in the biosynthetic pathway may also determine the rhythmicity of downstream volatiles emission under circadian control (Figure 2). In snapdragon flowers, rhythmic changes in the flux through the methylerythritol phosphate (MEP) pathway were shown to be controlled by the endogenous clock, which determined the rhythmicity of terpenoids emission [45]. In addition, the transcription levels of the gene encoding 1-deoxy-d-xylulose-5-phosphate synthase (DXPS), which catalyzes the formation of DXP, showed a diurnal oscillation pattern that strongly correlated with the rate of the MEP pathway (Figure 2). In maize, the transcript levels of *ZmLOX10*, which encodes 13-lipoxygenase, were shown to be strictly regulated by the circadian clock, with maximum expression coinciding with peak photosynthetic activity [46]. Since ZmLOX10 can mediate green leaf volatiles (GLVs) emission, there may be a mechanistic link between clock-regulated expression of *ZmLOX10* and circadian GLV emission [30].

In some cases, the efficiency of volatiles emission depends on the availability of substrates for the final step of volatile formation, especially when the enzymes responsible for the final reaction have broad substrate specificity [47,48]. The rhythmic production and emission of VPBs such as methyl benzoate in petunia flowers were shown to be regulated primarily by the level of the substrate, benzoic acid. In turn, the benzoic acid level was regulated by the expression of genes encoding key enzymes in its biosynthesis [36]. Although a close relationship between substrate availability and the rhythmic emission of volatiles has been proven in some plant species [36,49], little is known about the molecular mechanisms underlying the inducible rhythmic emission of volatiles. The formation of volatiles may depend not only on substrate availability and the requisite biosynthetic enzymes, but also on transcription factors. Therefore, there may be upstream signaling parts regulating the rhythmic emission of volatiles. Two morning components, circadian clock associated 1 (CCA1) and late elongated hypocotyl (LHY), and two evening components, the response regulator/CCT-domain protein timing of cab expression 1 (TOC1) and zeitlupe (ZTL), make up the main oscillator at the core of the plant clock [50,51]. The expression levels of *CCA1/LHY* and *TOC1* oscillate at high temperatures, and isoprene oscillates at that high temperature as well, indicating that there may be an endogenous oscillator playing a role in controlling rhythmic isoprene emission [38]. Further investigations are needed to verify the correlation between the CCA1/LHY-TOC1 molecular oscillator and the rhythmic emission of isoprene. Furthermore, PhLHY was shown to regulate the daily expression patterns of VBP pathway genes and the production of floral volatiles in petunia. Fenske et al. reported that *PhLHY* directly set the timing of floral volatile emission by restricting VBP genes expression to the evening in petunia [52]. In *Nicotiana attenuata*, silencing *NaLHY* and *NaZTL* strongly altered circadian rhythms in flowers [53]. Besides these two genes, another circadian clock gene, *NaTOC1*, also had an influence on the floral circadian oscillations for *Nicotiana attenuata* [51]. These studies indicated that conserved clock components in *Nicotiana attenuata* coordinate the floral rhythms [51,53].

## 5. Potential Impact of Other Factors on Circadian Rhythmic Emission of Plant Volatiles

During plant growth, many external/environmental factors such as biotic factors (insects and microorganisms) and abiotic factors (light, temperature) affect the emission of plant volatiles. Most studies on plant volatiles emission have focused on one or multiple external stresses. Less attention has been paid to the effects of interactions between external stresses and the endogenous circadian clock on the emission of plant volatiles.

The emission of β-ocimene from lima bean (*Phaseolus lunatus*) is an example of the effect of an interaction between biotic stress (insect attack) and the circadian clock on plant volatiles emission. Arimura et al. investigated whether herbivore-induced emission of β-ocimene from *P. lunatus* leaves was controlled by the circadian clock [54]. They demonstrated that herbivore-induced β-ocimene emission was not controlled by the circadian clock, but was strictly linked to light. However, the expression of *PlOS*, which encodes ocimene synthase, was shown to be regulated by the endogenous circadian clock, jasmonic acid, and light. In maize, *ZmLOX10* was shown to be induced by jasmonate and herbivores for defense against insect attack [30]. *ZmLOX10* was regulated by the circadian clock at a transcriptional level [46]. Clock-regulated expression of *ZmLOX10* was proposed to be a mechanistic link for the circadian emission of GLVs, but there was no direct evidence for the circadian emission of herbivore-induced GLVs. In some cases, therefore, genes involved in the formation and emission of herbivore-induced plant volatiles are regulated transcriptionally by the circadian clock, but this does not necessarily lead to circadian emission of herbivore-induced plant volatiles.

Van Moerkercke et al. proposed that the circadian clock is responsible for rhythmic volatiles emission in petunia that produce VPBs as the major volatile [55], and the circadian clock gene has been verified as a direct regulator [52]. Cheng et al. further demonstrated that VPBs showed stable rhythmic emission, regardless of different light wavelengths (for example, blue light, red light), continuous light or darkness, or different temperatures in petunia [9]. The endogenous circadian clock was therefore proposed to be the main factor regulating the rhythmic emission of VPBs in petunia. This is an example of the interaction between abiotic stress (light or temperature) and circadian clocks on plant volatiles emission reported for the flowers of petunia [9]. The endogenous circadian clock also influenced the expression of structural genes involved in the upstream biosynthetic pathway of VPBs, but did not affect those of structural genes involved in the downstream pathway or VPB-related regulators. Taken together, these results showed that the endogenous circadian clock played a dominant role in the rhythmic emission of VPBs from petunia flowers. Increasing ambient temperature led to a decrease in phenylpropanoid-based floral scent production in two *Petunia × hybrida* varieties, P720 and Blue Spark, acclimated at 22/16 °C or 28/22 °C (day/night) [56]. Nonetheless, an increase in temperature, even to 38 °C, did not break the circadian rhythmic emission of isoprene from oil palm [38].

## 6. Concluding Remarks and Perspectives

In this review, we summarize the current knowledge of circadian clock-mediated rhythmic emission of plant volatiles, although few studies have focused directly on this topic. The huge variety of volatiles emitted from plants and their complex and diverse emission patterns suggest that there is still much to learn. Several important questions should be addressed in future studies:

1 Not all plant volatiles show rhythmic emission regulated by the circadian clock. In addition, not all plants and not all plant tissues show such rhythmic emissions. Why are some plant volatiles rhythmically released and others not? Why is the emission of some plant volatiles controlled by endogenous circadian mechanisms while the emission of others is controlled by external factors such as light or temperature?
2 Many studies on the regulation of the rhythmic emission of plant volatiles by the circadian clock focus on downstream pathways or the final step in volatile biosynthesis. Is there a unified mechanism of action for the circadian clock to mediate the rhythmic emission of plant volatiles? What are the upstream signaling events that control circadian clock-mediated rhythmic emission?
3 In most cases, external factors such as insect attack, high temperature, or light readily induce the transient emission (in minor cases, rhythmic emission) of plant volatiles, some of which can protect plants. Why do plants regulate rhythmic emission of plant volatiles through the endogenous circadian clock? What are the benefits for plant growth and development? Besides ecological functions such as interaction with insects, do volatiles have physiological functions in plants?
4 Plants are exposed to many environmental factors/stresses. Are there interactions between these environmental factors and regulation of the endogenous circadian clock? If endogenous circadian clock regulation is relatively independent, how do plants maintain the stability of endogenous circadian clock regulation?

## Figures and Tables

**Table 1:** List of plant volatiles rhythmically emitted under the control of the circadian clock.

Volatile	Plant	Emission Level	Ref.
Volatile phenylpropanoids/benzenoids			
l-Nitro-2-phenylethane	*Stephanotis floribunda*	-	[17]
Methyl benzoate	*Antirrhinum majus*	0.6–1.9 μg/g FW	[36]
	*Nicotiana suaveolens*	-	[36]
	*Petunia × hybrida*	-	[36]
	*Nicotiana sylvestris*; *Nicotiana suaveolens*	-	[37]
Benzyl alcohol	*Nicotiana sylvestris*; *Nicotiana suaveolens*	-	[37]
Methyl salicylate	*Nicotiana suaveolens*	-	[37]
	*Stephanotis floribunda*	-	[42]
Volatile terpenes			
Isoprene	*Elaeis guineensis*	-	[38]
	*Populus × canescens*	nearly 100–1600 nmol/g FW·s	[39]
β-Pinene	*Artemisia annua*	-	[43]
β-Ionone	*Petunia × hybrida*	nearly 22–25 pg/g FW·h	[40]
(*E*)-β-Ocimene	*Antirrhinum majus*	nearly 0.2–3 μg/flower·h	[41]
Myrcene	*Antirrhinum majus*	nearly 0.05–0.8 μg/flower·h	[41]
Linalool	*Hoya carfiosa*	-	[44]

Ref., reference; FW, fresh weight; ‘-’, the volatile emission level was not reported in the reference.

## Abbreviations

CCA1	Circadian clock associated 1
CCD	Carotenoid cleavage dioxygenase
DXP	1-Deoxy-d-xylulose-5-phosphate
DXPS	1-Deoxy-d-xylulose-5-phosphate synthase
GLVs	Green leaf volatiles
LHY	Late elongated hypocotyl
LOX	Lipoxygenase
MEP	Methylerythritol phosphate
SAMT	Salicylic acid carboxyl methyltransferase
ISPS	Isoprene synthase
TOC1	Timing of cab expression 1
VPBs	Volatile phenylpropanoid/benzenoid
